# Hybrid microneedle arrays for antibiotic and near-IR photothermal synergistic antimicrobial effect against Methicillin-Resistant *Staphylococcus aureus*

**DOI:** 10.1016/j.cej.2023.142127

**Published:** 2023-04

**Authors:** Jill Ziesmer, Justina Venckute Larsson, Georgios A. Sotiriou

**Affiliations:** Department of Microbiology, Tumor and Cell Biology, Karolinska Institutet, SE-171 77 Stockholm, Sweden

**Keywords:** Skin patch, MRSA, Thermotherapy, Local delivery, Gold nanoparticles, Combination treatment

## Abstract

The rise of antibiotic-resistant skin and soft tissue infections (SSTIs) necessitates the development of novel treatments to improve the efficiency and delivery of antibiotics. The incorporation of photothermal agents such as plasmonic nanoparticles (NPs) improves the antibacterial efficiency of antibiotics through synergism with elevated temperatures. Hybrid microneedle (MN) arrays are promising local delivery platforms that enable co-therapy with therapeutic and photothermal agents. However, to-date, the majority of hybrid MNs have focused on the potential treatment of skin cancers, while suffering from the shortcoming of the intradermal release of photothermal agents. Here, we developed hybrid, two-layered MN arrays consisting of an outer water-soluble layer loaded with vancomycin (VAN) and an inner water-insoluble near-IR photothermal core. The photothermal core consists of flame-made plasmonic Au/SiO_2_ nanoaggregates and polymethylmethacrylate (PMMA). We analyzed the effect of the outer layer polymer, polyvinyl alcohol (PVA) and polyvinylpyrrolidone (PVP), on MN morphology and performance. Hybrid MNs produced with 30 wt% PVA contain a highly drug-loaded outer shell allowing for the incorporation of VAN concentrations up to 100 mg g^−1^ and temperature increases up to 60 °C under near-IR irradiation while showing sufficient mechanical strength for skin insertion. Furthermore, we studied the combinatorial effect of VAN and heat on the growth inhibition of methicillin-resistant *Staphylococcus aureus* (MRSA) showing synergistic inhibition between VAN and heat above 55 °C for 10 min. Finally, we show that treatment with hybrid MN arrays can inhibit the growth of MRSA due to the synergistic interaction of heat with VAN reducing the bacterial survival by up to 80%. This proof-of-concept study demonstrates the potential of hybrid, two-layered MN arrays as a novel treatment option for MRSA-associated skin infections.

## 1 Introduction

The skin is the largest organ of the human body and preliminarily functions to protect the body from the environment. While the skin is cultivated by commensal microorganisms [1,2], the colonization by pathogenic bacteria can cause skin and soft tissue infections (SSTIs) [3]. An estimated 7–10% of hospitalized patients are affected by SSTIs, with rising incidence in the past two decades [4–6]. The increase in SSTIs is not only a health and cosmetic problem for patients, but it is also an economic burden with an expected $15 billion in spending in the U.S. in 2012 [6]. Generally, SSTIs are treated with antibiotics, many of which are administered via the oral or parental route [7]. However, the systemic administration of antibiotics may suffer from multiple shortcomings, such as (i) lower relative antibiotic concentrations at the infection site [8,9] and (ii) exposure of healthy microbiota to antibiotics. Such shortcomings can lead to the development of antibiotic resistance, as bacteria are exposed to subtherapeutic concentrations [10]. Furthermore, exposure of healthy bacteria to antibiotics increases the prevalence of antibiotic-resistant strains [11,12].

Multiple attempts to overcome the shortcomings associated with systemic antibiotic administration in dermatology have been reported in the literature. The development of targeted drug delivery systems and local delivery routes allows for an increase in antibiotic concentration at the site of infection while reducing off-target exposure to healthy tissue and microbiota [13–15]. As such, microneedle (MN) arrays have been introduced as potential intra- and transdermal delivery platforms for antibiotics [16,17]. MN arrays are patches comprising multiple MNs that can be applied to the skin with minimal pain and training [18,19]. MNs made with dissolvable polymers have been widely researched in the field of drug delivery because of their ability to deliver their cargo upon dissolution in the humid environment of the skin, thus enabling high local drug concentrations [20,21]. Dissolvable MN arrays have been successfully used to deliver the antibiotics gentamicin [22,23], tetracycline [24], chloramphenicol [25], polymyxin [26], cephalexin [27], doxycycline [28,29], clindamycin [30], and vancomycin (VAN) [31,32] inside or across the skin. It has been shown that the delivery of VAN into the local skin environment in mice can be increased 500-fold when MNs are used instead of intravenous injection [32]. Furthermore, VAN delivered with MN arrays retained antibacterial activity after delivery and resulted in antibacterial growth inhibition of methicillin-resistant *Staphylococcus aureus* (MRSA) in ex vivo porcine models [31].

The combination of antibiotics with nanoparticles (NPs) may improve the antibacterial effect through additive or synergistic antibacterial effects by chemically or physically weakening the bacteria [33,34]. Such mechanisms to weaken bacteria chemically or physically with NPs involve thecreation of reactive oxygen species,[35,36] anti-bacterial ions [37], or elevated temperatures [38,39]. The use of thermotherapy is widely described in literature; the exposure of bacteria to temperatures of 45–55 °C has a direct antimicrobial activity, resulting in cellular immune responses and an alteration of cytokine levels [40,41]. Photothermal NPs from inorganic materials such as Ag [42] and Au [43] allow for thermotherapy under irradiation with near-IR light, rendering them suitable for application in human tissue [44]. Au NPs are particularly interesting for potential clinical translation because of their high photothermal efficiency, chemical stability, and low cytotoxicity [45]. Furthermore, Au NPs have been successfully employed in combination therapy with antibiotics against (antibiotic-resistant) bacterial strains [46].

Hybrid MN arrays containing both pharmaceutical drugs and photothermal NPs are attractive drug delivery platforms for the local treatment of skin diseases. Such hybrid MN arrays have been predominantly investigated for the treatment of skin cancers, and the photothermal agents employed in these MN arrays range from inorganic and metal NPs such as LaB_6_ [47,48], Au nanorods [49], Au nanocages [50], and Si-coated Au [51], to organic agents such as melanin [52] and indocyanine green [53–55]. In addition to the enhancement of chemo-therapy, photothermal NPs made from Prussian blue [56,57], Bi nano-dots [58], and Cu_7_S_4_ [59] were successfully used for the controlled melting of polymeric MNs to allow tunable metformin release for the treatment of diabetes. Furthermore, in recent years, several studies have investigated the antibacterial effect of such hybrid MNs for application in infections [60,61] and wounds [62]. However, all of these MN arrays deliver the photothermal agent into the skin, and since the long-term biosafety of intradermal NPs deposition and the resulting bio-accumulation is not yet completely established, it would be advantageous to avoid the delivery of NPs into the human body. We have recently published an article describing the fabrication of non-dissolvable photothermal MNs arrays that reduce such intradermal NPs deposition [63]. However, it would be advantageous to combine the photothermal therapy from MN arrays with drug delivery for synergistic treatments.

Here, we present a proof-of-concept study for the development of hybrid, two-layered MN arrays for the delivery of the antibiotic VAN from a water-soluble outer layer, and heat treatment from a water-insoluble, photothermal inner core. The fabrication of the photothermal core was based on a previous study by our group, in which we optimized the incorporation of photothermal Au/SiO_2_ (4 wt% SiO_2_) NPs active in the near-IR region into the water-insoluble polymer polymethylmethacrylate (PMMA) [63]. For the fabrication of the outer layer, we explored two different water-soluble polymers, namely polyvinyl alcohol (PVA) and polyvinylpyrrolidone (PVP), at various concentrations and analyzed how the choice of the polymer allows for the control of the MN design, which in turn changes the drug release and photothermal effect. Finally, we studied the potential therapy of MRSA infections through a synergistic interaction between VAN and heat from our hybrid MN arrays.

## 2 Experimental section

### 2.1 Synthesis of Au/SiO_2_ (4 wt% SiO_2_) nanoparticle (NP) powders

Plasmonic Au/SiO_2_ (4 wt% SiO_2_) nanoaggregates were synthesized by flame spray pyrolysis [64]. Briefly, acetonitrile (99.8%, Sigma-Aldrich) and 2-Ethyl hexanoic acid (2-EHA, 99%, Sigma-Aldrich) were mixed in a ratio 1:1 and 0.1 M gold acetate (99%, Alfa Aesar) was dissolved in the solvents at 110 °C for 1.5 h under reflux. After cooling down for 5 min, hexamethyldisiloxane (HDMSO, ≥98%, Sigma-Aldrich) was added to obtain a final nominal concentration of 4 wt% SiO_2_. A pilot flame with flow rates of 3.2 and 1.5 L min^−1^ oxygen/methane (>99.5%, Strandmöllen AB/ >99.5%, AGA Gas AB) was ignited, and the liquid precursor was combusted by feeding through a capillary at 10 mL min^−1^ and dispersion at 3 L min^−1^ oxygen. The NPs were collected as powders downstream on a glass fiber filter (Type GF6, Hahnemühle) via vacuum suction (Mink MM 1144 BV Busch).

### 2.2 Fabrication of hybrid MNs

Hybrid MNs were fabricated by a two-step casting method using MN molds (B200, H600, Micropoint Technology). First, aqueous solutions of desired weight percentages of polyvinyl alcohol (PVA, 87–89% hydroxylation, MW 12–23 k, Sigma-Aldrich) or polyvinyl pyrrolidone (PVP, MW 360 k, Sigma-Aldrich) were prepared. PVA was dissolved in water by stirring for 30 min at ~ 22 °C, 30 min at 80 °C, and > 5 h at 60 °C. An aqueous solution of PVP was prepared by mixing with a spatula at ~ 22 °C. In the case of dye- or drug-loaded hybrid MNs arrays, desired concentrations of sulforhodamine B (SR, 75% dye content, Sigma-Aldrich) or VAN hydrochloride (Sigma-Aldrich), respectively, were mixed with a spatula in an aqueous polymer solution for 1 min, sonicated in a bath sonicator for 5 min, centrifuged for 2 min at 10,000 rpm, and resuspended for 1 min. The aqueous polymer blend (50 mg) was added to the MN mold, centrifuged for 15 min at 3500 rpm, the surplus was removed with a spatula, and the molds were dried for 15 min at 50 °C. Another 50 mg of polymer blend was added, and the molds were centrifuged, surplus removed, centrifuged again, and dried. Second, water-insoluble polymethyl methacrylate (PMMA, MW 120 k, Sigma-Aldrich) solutions were prepared by dissolving PMMA in ethyl lactate (≥98%, Sigma-Aldrich) under stirring at 150 °C for 1.5 h. The NP-loaded core was produced by sonicating the desired concentration of NPs in 10 wt% PMMA in a bath sonicator for 10 min. The NP-PMMA blend (50 mg) was added to the MN mold and centrifuged as described above, and the surplus was removed using a spatula. Finally, the support layer was cast by adding 30 mg of a 30 wt% PMMA solution to the MN mold, centrifuging for 10 min, and drying overnight. The final MN arrays were peeled off the mold using tweezers and imaged using bright-field or fluorescence microscopy (Leica MZ10) or scanning electron microscopy (SEM, Phenom Pharos SEM, Thermo Fisher Scientific).

### 2.3 Mechanical testing

Mechanical testing was performed using a texture analyzer (TA.XT plusC, Stable Micro System) as described previously [65,66]. The MN arrays were placed on a cylinder probe using double adhesive tape and compressed for 30 s at 32 N. The cylinder probe was operated at 1 mm s^−1^ pre-, post-, and test speeds. MNs were imaged with bright-field microscopy before and after compression, and the height reduction was analyzed using ImageJ (US National Institute of Health) [67].

### 2.4 In-air photothermal effect

The photothermal effect of the MNs under 808 nm laser irradiation was analyzed by measuring the heat response from above the sample using a thermal imaging camera (Testo 871, Testo). The laser was operated by connecting a fiber-coupled diode laser to a top-hat diffuser (Laser Century), resulting in a square laser area. The final laser power was quantified using a thermal optical power meter (Thorlabs S425C), and the distance between the diffuser and MN arrays was set to a beam intensity of 1 W cm^−2^. The temperature increase was quantified based on 6 × 6 pixels around the hotspots of the thermal images.

### 2.5 Drug quantification

Hybrid MN arrays were prepared as described above with PVP (15 or 30 wt%) or PVA (15, 20, 30, or 40 wt%) containing 10 mg g^−1^ VAN. The MN arrays were completely dissolved in phosphate-buffered saline (PBS, 0.1 M, pH 7.4) for 30 min under shaking and the samples were diluted to VAN concentrations between 0.05 and 2 μg ml^−1^. The samples were analyzed by reversed-phase high-pressure liquid chromatography (HPLC, 1260, Agilent), as reported previously [31]. Briefly, a mobile phase consisting of NH_4_H_2_PO_4_ (50 mM, pH 4) and acetonitrile at a ratio of 92:8 was fed at 1 mL min^−1^ into a Nucleosil C18 column (5 μm particle size, 15 cm length, 4.6 mm inner diameter, Sigma-Aldrich). The column temperature was maintained at 40 °C, injection volume was 20 μL, run time 11 min, and detection was performed at 220 nm reference to 360 nm. The concentration of VAN was calculated using linear regression of the peak area to known VAN concentrations in the range of 0.05 to 2 μg ml^−1^. For VAN release studies, hybrid MN arrays prepared with 100 mg mL^−1^ were exposed to 1 mL water and 100 µL samples were withdrawn at 0.5, 1, 2, 3, 5, 10, 20, 30, and 60 min and analyzed for their VAN content via HPLC.

### 2.6 Excised porcine skin studies

Excised pork skin was obtained from stillborn piglets collected at a local farmer which is except from ethical approval. Hybrid MN arrays were applied with thumb pressure to the skin tissue and irradiated with a near-IR laser from the backing layer at 1 W cm^−2^ at 808 nm. Analysis of the thermal images was conducted as outlined above. After insertion into skin the MN arrays were analyzed via HPLC for the intradermally delivered amount of VAN. Skin tissue were shock-frozen in liquid nitrogen, embedded in cryotome medium and 8 µm tissue sections were performed and imaged.

### 2.7 Antibacterial studies

Antibacterial studies were performed using MRSA (USA 300). Liquid cultures were prepared by inoculating Luria broth (LB) and incubating overnight at 37 °C shaking at 220 rpm. The optical density at 600 nm (OD_600nm_) was adjusted to 0.01 either in PBS or LB medium. To analyze the synergistic effect of VAN and heat, bacterial solutions in PBS were heated in a heat block for various times to reach different temperatures in the bacterial liquid. After heat treatment, 200 μL of bacterial solution was diluted with 200 μL of Terrific Broth (TB) medium and mixed with concentrated stocks of VAN to obtain final VAN concentrations between 0 and 0.75 μg ml^−1^. After vortexing, 200 μL of bacterial samples was transferred to honeycomb multiwell plates, which were incubated at 37 °C under regular shaking with measurements of the OD_600nm_ every 10 min for 24 h in a Bioscreen C instrument (Growth curves OY, Turku, Finland). Growth inhibition was calculated by dividing the OD_600nm_ of the treated samples by that of the control. Loewe additivity was analyzed using Combenefit software [68]. For antibacterial testing of the hybrid MNs, MN arrays containing varying concentrations of VAN (0, 0.5, 1, and 10 mg g^−1^) were fabricated as described above. Bacterial solution (200 μL) in LB at an OD_600nm_ of 0.01 were added to the MN arrays in a well plate. MN arrays were irradiated with an 808 nm laser at 1 W cm^−2^ for 15 min from the top and imaged with a thermal camera from the bottom. The bacterial samples were incubated for 4 or 24 h at 37 °C with shaking at 220 rpm. After incubation, samples were centrifuged for 2 min at 14,000 × g and a ten-fold dilution series was prepared. Dot spots on Luria agar containing 10 μL diluted samples were prepared, incubated overnight, quantified, and imaged.

## 3 Results and discussion

### 3.1 Fabrication of hybrid microneedle arrays

**Fig. 1:**
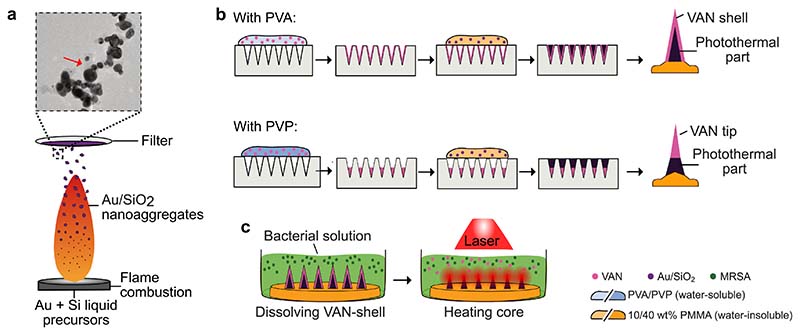
Schematic illustration of the fabrication process of two-layered hybrid MN arrays. (a) Illustration of the aerosol synthesis of photothermal Au/SiO_2_ (4 wt% SiO_2_) nanoaggregates. A liquid precursor containing Au and Si is combusted in a methane/oxygen supported flame and the nanoaggregate-containing aerosol is filtered downstream via vacuum-aided filtration. The nanoaggregate filter cake is collected, and the powder is used for further fabrication of the hybrid MN arrays. The insert shows a TEM image of the Au/SiO_2_ aggregates, the SiO_2_ layer is highlighted with the red arrow. (b) Schematic of the fabrication steps for production of two-layered hybrid MN arrays using either PVA or PVP for the drug-containing, water-soluble outer layer and PMMA for the nanoaggregate-containing, water-insoluble core. (c) Final synergistic antibacterial action of hybrid MN arrays by (i) release of the antibiotic VAN through dissolution of the water-soluble outer layer followed by (ii) photothermal heating under near-IR laser radiation. (For interpretation of the references to color in this figure legend, the reader is referred to the web version of this article.)

Fig. 1 illustrates the fabrication steps for the two-layered hybrid MN arrays composed of an outer layer containing the antibiotic VAN and a photothermal core loaded with plasmonic Au/SiO_2_ (4 wt% SiO_2_) NPs. Photothermal Au nanoaggregates were produced via aerosol synthesis, and we selected 4 wt% SiO_2_ based on our previous reports showing an improved plasmonic coupling in the near-IR spectrum at this dielectric spacer content [63]. Fig. 1a shows a simplified schematic of aerosol synthesis via flame spray pyrolysis, in which a liquid precursor containing Au and Si is combusted to form Au/SiO_2_ nanoaggregates that are collected by vacuum-aided filtration. The inset of Fig. 1a (see also supporting information (SI), Fig. S1a) shows a TEM image of the Au/SiO_2_ nanoaggregates, indicating the presence of a thin SiO_2_ layer (red arrow) that effectively separates the individual Au NPs, thus acting as a spacer. Furthermore, analysis of the specific surface area via N_2_ absorption and the measurement of the crystalline size from XRD diffractogram as summarized in Fig. S1b demonstrates high reproducibility among production batches. The UV/Vis analysis of dispersed NPs in water in Fig. S1c confirms the absorption in the near-IR spectrum in line with our previous report [63]. To fabricate two-layered hybrid MN arrays, we utilized a water-soluble polymer (PVA or PVP) as the matrix for the outer layer and a water-insoluble polymer (PMMA) as the core material. Fig. 1b shows the fabrication steps involved in the production of hybrid MN arrays, in which (i) the water-soluble, drug- or dye-loaded polymer is concentrated into the MN cavities of MN molds by centrifugation, followed by (ii) centrifugation of NP-loaded PMMA to form the photothermal cores, and (iii) addition of blank PMMA as the backing layer to detach the hybrid MN array from the mold. The choice of the water-soluble polymer controlled the shape of the outer MN layer; as such, PVA formed a thin shell in the MN cavities, whereas PVP was concentrated at the tip of the cavity, as will be discussed later. Ultimately, we aimed to study the antibacterial effect of the hybrid MN arrays by treating planktonic MRSA with the hybrid MNs arrays under near-IR irradiation, as illustrated in Fig. 1c.

**Fig. 2:**
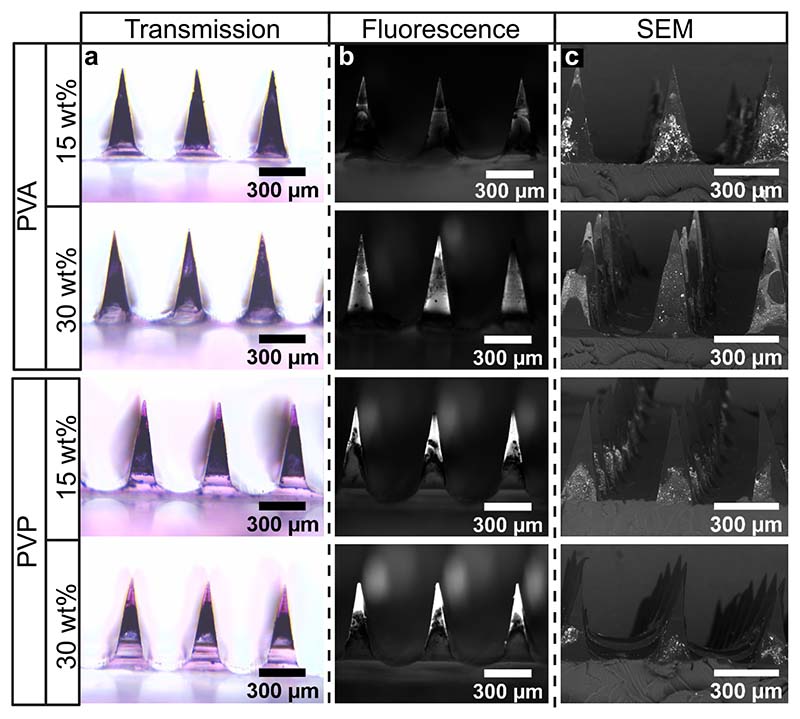
Microscopy images of two-layered hybrid MN arrays loaded with the dye sulforhodamine for two different water-soluble polymers (PVA and PVP) and two polymeric weight concentrations (15 and 30 wt%). (a) MNs are shown imaged with bright field microscopy to visualize the pink dye and the dark Au/SiO_2_ (4 wt% SiO_2_) nanoaggregates. (b) Fluorescence microscopy images are shown to visualize the water-soluble layer via the fluorescence signal from the dye (in white). (c) The MN arrays were also imaged via SEM to highlight the nanoaggregate-loaded layer (in white). (For interpretation of the references to color in this figure legend, the reader is referred to the web version of this article.)

To analyze the effect of the choice and concentration of the polymer on the formation of the outer water-soluble layer, we fabricated hybrid MN arrays utilizing 15 or 30 wt% PVA or PVP solution loaded with the pink, fluorescent dye sulforhodamine. Fig. 2 shows bright field microscopy, fluorescence microscopy, and SEM images of single MNs under four different conditions. The MN arrays in the bright-field microscopy images (Fig. 2a) appear black, indicating the presence of Au NPs. The pink dye can only be distinguished in MN arrays fabricated with PVP, indicating the formation of a water-soluble MN tip that increases in size at higher PVP concentrations. To further validate the presence of the dye in the hybrid MN arrays, we obtained fluorescence microscopy images (Fig. 2b). The dye-loaded outer layer (bright signal) can now be detected for MN arrays produced by either PVA or PVP. Utilizing PVA resulted in the presence of a fluorescent signal around the complete MN, indicating the formation of a shell. The formation of a tip was again verified for MN arrays produced with PVP, in agreement with the bright-field microscopy results. Finally, to analyze the distribution of the Au nano-aggregates in the MNs, we obtained the SEM images shown in Fig. 2c with high-density elements such as Au appearing bright. When utilizing PVA as the polymer to fabricate the outer layer, the presence of Au NPs can be distinguished on the surface of the complete MNs, indicating that the shell formed by PVA may be porous or partially combined with the photothermal composite, thus allowing water-insoluble polymers loaded with Au to enter the outer layer during fabrication. In contrast, the tip formed when using PVP did not indicate the presence of Au NPs in the MN area occupied by the dye-loaded polymer. Instead, the Au NPs were present below the dye-loaded tip in the core of the MN, which seemed to decrease in size with increasing PVP concentration. A similar tip formation to that observed with PVP was reported in a study by Than *et al.*, in which two-layered MNs with an outer layer consisting of cross-linked methacrylated hyaluronic acid formed a distinct layer outside of the MNs [69]. The formation of a tip or shell depending on whether PVP or PVA is used here may be due to differences in the molecular weight, hydrophobicity, or degree of hydrolysis of the polymers used, which warrants more detailed investigations in future studies. To summarize, we demonstrate the successful fabrication of two-layered hybrid MNs, in which the selection of the outer water-soluble polymer allows for the control of the final MN design.

### 3.2 Drug loading in hybrid MN arrays

**Fig. 3:**
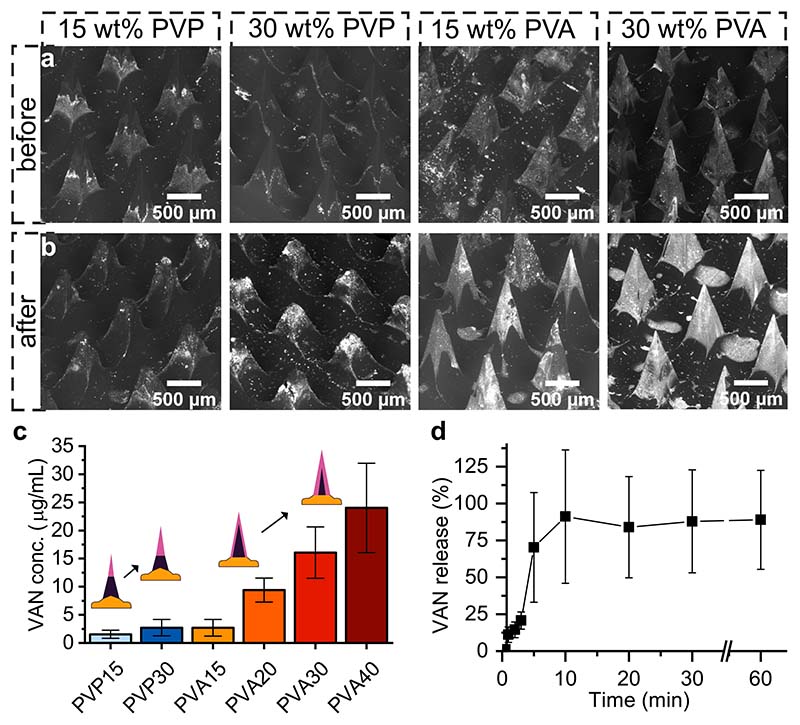
Drug loading of VAN in hybrid MN arrays. SEM images of hybrid MN arrays (a) before and (b) after dissolution in PBS for different polymers and weight percentages. (c) Quantification via HPLC of VAN released into PBS from hybrid MN arrays produced with 10 mg g^−1^ VAN in different polymers for different weight percentages, n = 3. Insert shows schematic illustration of the influence of increasing weight percentages of PVP or PVA, respectively, on the formation of the VAN-loaded layer in the hybrid MN arrays. (d) VAN release over time from hybrid MN arrays produced with 30 wt% PVA, n = 3.

Drug solubility and diffusion within multilayered MNs depend on the choice of polymers [31,70]. Thus, to study the effect of the polymer on drug loading in hybrid MN arrays, we fabricated MNs with PVP and PVA at varying polymer concentrations and loaded them with 10 mg g^−1^ of the antibiotic VAN. We then completely dissolved the MNs for 30 min in PBS and quantified the amount of VAN released via HPLC using a previously established protocol [31]. Fig. 3a and b show SEM images at the same brightness and contrast settings of hybrid MN arrays produced with 15 or 30 wt% PVP and PVA before (Fig. 3a) and after (Fig. 3b) complete dissolution of the water-soluble outer layer (bright-field microscopy images of the same samples are shown in Supporting Information, Fig. S2). The sharp tip of the hybrid MN arrays produced with PVP disappeared after dissolution, and the remaining water-insoluble, NP-loaded PMMA core reduced in size with increasing PVP concentration, confirming the increasing tip size at high PVP concentrations.

For the MNs produced with PVA, we detected complete MNs with sharp tips, even after dissolution. The bright signal of high-density elements, such as Au, in the SEM images increased after dissolution, indicating that the water-soluble PVA shell dissolved and the NP-loaded core remained. While an SEM signal from Au can be distinguished before MN dissolution, the signal increased significantly after dissolution, further validating the above hypothesis that the outer, water-soluble PVA shell only partially enclosed the water-insoluble, NP-loaded polymer. To quantify the amount of VAN released from the hybrid MNs, we analyzed the solution after the complete dissolution of the hybrid MNs using HPLC, and the results are shown in Fig. 3c (raw data in SI, Fig. S3). On the one hand, we found that using PVA instead of PVP allows for the loading of higher amounts of VAN, with a maximum loading of 24 or 3 μg VAN per array, respectively. The increased loading capacity of VAN in the hybrid MNs fabricated with PVA rather than PVP may be explained by the reduced dissolution of VAN into the water-insoluble core when using PVA. As shown in our previous study, VAN has a lower solubility in PVP than PVA, thus allowing more drug to diffuse into the water-insoluble core of the hybrid MN, effectively decreasing the deliverable amount of drug when using PVP [31]. On the other hand, we conclude from the results in Fig. 3c that increasing the polymeric weight percentage increases the drug-loading capacity when using PVP and PVA, which may be caused by a decrease in VAN diffusion into the water-insoluble core. This is in agreement with the literature showing that a higher polymeric weight percentage increases polymer-drug interactions, which in turn decreases the diffusion coefficient of drugs [71]. Finally, we measured the VAN release in water over time from hybrid MNs arrays fabricated with 30 wt% PVA in Fig. 3d, showing that above 90 % of VAN is released from the MNs within 10 min. To summarize, we successfully fabricated hybrid MNs arrays consisting of a drug-loaded outer layer that selectively dissolved and released the drug, while the photothermal agent remained in the water-insoluble core of the MN array.

### 3.3 Photothermal performance of hybrid MN arrays

**Fig. 4:**
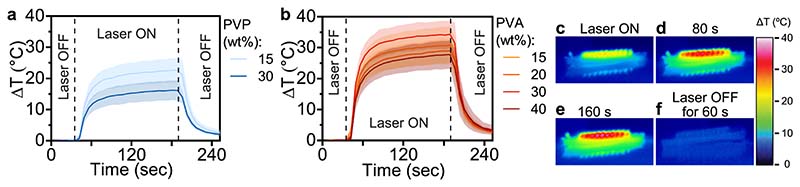
Photothermal heating of hybrid MN arrays (prepared with 10 mg g^−1^ VAN and 20 mg g^−1^ Au/SiO_2_ NPs) at 808 nm laser radiation in air. (a) Temperature increase of hybrid MN arrays prepared with (a) 15 or 30 wt% of PVP, or (b) 15, 20, 30, and 40 wt% PVA, n = 3. (c-f) Thermal images obtained during laser irradiation of hybrid MN array prepared with 30 wt% PVA at 0, 80, 160, or 240 s, respectively.

We hypothesized that the choice and concentration of the water-soluble polymer not only influences the drug loading of the hybrid MN arrays but also the photothermal effect. We fabricated hybrid MN arrays with PVP or PVA at different weight percentages to analyze their photothermal effect using a thermal camera under irradiation with a near-IR laser at 808 nm. The average temperature increase of 36 pixels around the center of the MN arrays is plotted as a function of time in Fig. 4a and 4b for MNs fabricated with PVP and PVA, respectively. The thermal images of the MN arrays were recorded for 30 sec before 3 min of laser irradiation followed by 1 min of recording the thermal images after end of irradiation. We found that using PVA instead of PVP to produce hybrid MN arrays resulted in a higher temperature increase. This may be caused by the formation of a PVA shell around the NP-loaded core, as opposed to a tip, when using PVP, thus allowing for the formation of a large NP-loaded MN surface. Furthermore, we found that increasing the concentration of PVP from 15 to 30 wt% decreased the photothermal effect due to the decreasing size of the photothermal core, as outlined above in Fig. 4a and 4b. However, increasing the concentration of PVA did not similarly decrease the photothermal effect, indicating that the increasing shell thickness did not negatively impact the photothermal effect of the NP-loaded core in the hybrid MN arrays produced with PVA. Importantly, for hybrid MN arrays fabricated with 30 wt% of PVA, we obtained a maximum temperature increase (ΔT_max_) of 34 °C after 2.5 min of laser irradiation at 1 W cm^−2^, which is similar [49,50] or better [72] than the photothermal effect of MNs in the literature. High-resolution thermal images of the MN arrays under near-IR irradiation depicted in Fig. 4c-f shows that the temperature increase is highest in the MN core, resulting in a ΔT_max_ of up to 40 °C demonstrating that a fine tuning of the irradiation is necessary to avoid thermal damage to the skin. The ΔT_max_ value achieved in air results in a sufficient temperature increase in the skin, as validated by ex vivo thermal imaging [63]. Overall, the target temperatures against bacterial infection in the skin are between 38 and 50 °C [41]. We are able to reach such targeted temperatures for laser irradiation of the MNs when applied from the top of the array facing the MNs or from the bottom facing the backing layer as shown in the SI, Fig. S4. Thus, the temperature increase achieved with our hybrid MN array may be sufficient for successful photothermal therapy of bacterial skin infections.

Based on the ideal drug loading, photothermal effect, and handling of the polymer solution, we selected hybrid MNs produced with 30 wt% PVA for the remainder of the study and explored the mechanical strength against compression for 30 s at 32 N (human thumb pressure) [65] using a texture analyzer, as reported previously [66]. Hybrid MNs fabricated with 30 wt% PVA were similar to or stronger than those fabricated with PVP or 15 wt% PVA (SI, Fig. S5 and Fig. S6). We explored the influence of NP and VAN loading on the mechanical strength of the MN arrays made with 30 wt% PVA and found that (i) increasing the NP concentration from 0 to 20 mg g^−1^ decreased the compression strength slightly (15% increase in height reduction), while (ii) increasing VAN concentration (0–100 mg g^−1^) did not seem to influence the strength of the MNs (SI, Fig. S5c,d). Importantly, the photothermal MN arrays reported in the literature, with similar height decreases as our hybrid MN arrays, are sufficiently strong to insert into the skin [66]. To further evaluate the potential application of our hybrid MN arrays for skin infections, we fabricated MNs using 30 wt% PVA with 20 mg g^−1^ NPs and 100 mg g^−1^ VAN loading and pierced it into excised full-thickness porcine skin using thumb pressure. In the SI, Fig. S7 shows tissue sections of the skin after MN array removal, indicating the successful breaching of the outer skin layer resulting in the formation of small puncture holes. This demonstrates the sufficient mechanical stability of the here prepared hybrid MN arrays allowing for synergistic skin treatments in a single MN application.

### 3.4 Synergistic effect of VAN and heat on MRSA

**Fig. 5:**
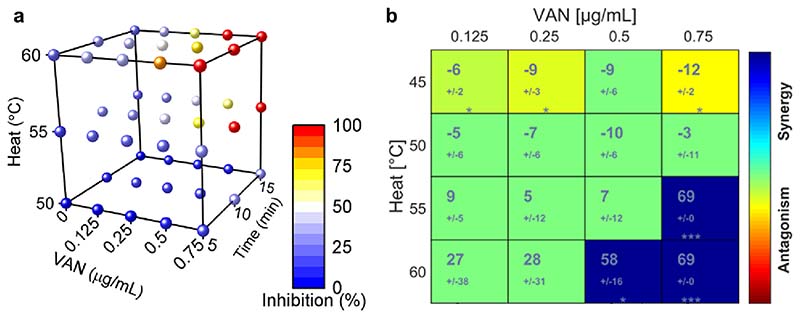
In vitro synergistic effect between heat and VAN on bacterial growth inhibition of MRSA. (a) Growth inhibition of MRSA 24 h after exposure to different temperatures (50–60 °C), for several treatment durations (5–15 min) and in presence of VAN at varying concentrations (0–0.75 μg mL^−1^). The color indicates the average growth inhibition for three replicates. (b) Calculation of combination effect of heat exposure for 10 min and VAN on the bacterial growth of MRSA after 24 h according to Loewe additivity. Three replicates were analyzed using the software tool Combenefit.

It has been extensively demonstrated that combining heat treatment with antibiotic therapy enhances the therapeutic efficiency of antibiotics [73–77]. However, the extent and effect of such synergistic treatments depend on the type of antibiotic and the bacterial species [40,78]. Therefore, we studied the effect of heat on the growth inhibition of MRSA by VAN at temperatures ranging from 45 to 60 °C. For this, we heated bacterial solutions with a heat block to the desired temperatures, added VAN, and monitored the optical density at 600 nm, which corresponds to bacterial growth over time. Fig. 5a shows the percentage growth inhibition after 18 h of MRSA for increasing VAN concentrations (0–0.75 μg mL^−1^), temperature (50–60 °C), and temperature treatment durations (5–15 min) (raw data in SI, Fig. S8). We found that growth inhibition increased as a function of temperature, VAN concentration, or heat treatment duration. Treatment with heat alone for 10 min resulted in a growth inhibition of up to 31% at 60 °C. However, this inhibition was drastically improved when VAN was added to the bacterial culture. Exposing MRSA to concentrations as low as 0.25 μg mL^−1^ VAN inhibited the growth by 48% after 10 min treatment at 60 °C, while the growth inhibition from this VAN concentration alone without heat treatment was only 3%. Furthermore, for the majority of VAN and heat combinations, increasing the heat treatment duration from 10 to 15 min did not drastically improve growth inhibition; thus, we selected a 10 min treatment as optimal heat therapy, comparable to co-therapies of heat and antibiotics tested in the literature [40].

To study the effect of the combination of antibiotics with heat, we obtained the Loewe additivity score using the software Combenefit [68] for selected VAN concentrations and temperatures for a 10 min treatment. The Loewe additivity scores between VAN and heat are shown in Fig. 5b, with values below –10, above –10, between 10 and 10, or around 0 indicating an antagonistic, synergistic, additive, or no effect, respectively. We found that heating above 45 °C enables additive growth inhibition of MRSA, whereas VAN concentrations and temperatures above 0.5 μg mL^−1^ and around 60 °C, respectively, are required to obtain a synergistic effect between the two treatments. However, heating above 50 °C in skin may induce protein denaturation and tissue coagulation [79]. Thus, we chose a treatment for 10 min at 45–55 °C as the ideal therapeutic heat window to enhance the antibiotic treatment of MRSA while minimizing the risk of harmful adverse effects.

### 3.5 Antibacterial efficiency of hybrid MN arrays

**Fig. 6:**
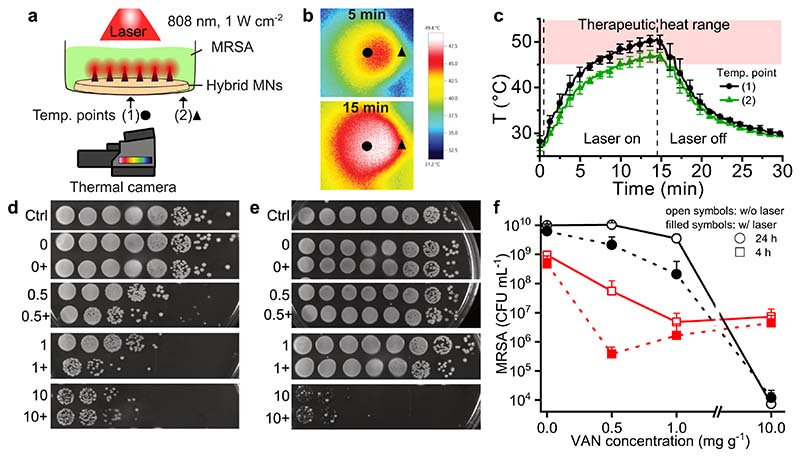
In vitro antibacterial performance of hybrid MN arrays against MRSA. (a) Schematic illustration of experimental set-up for photothermal heating of hybrid MNs arrays in liquid MRSA culture under NIR irradiation at 808 nm and 1 W cm^−2^. Indication of location of temperature measurement points (1, triangle) and (2, diamond) at the center or edge of the sample well, respectively. (b) Thermal images obtained after 5 and 15 min depicting the measurement points. (c) Temperature profile over 30 min of measurement points in the bacterial solution for NIR irradiation of 15 min. (d,e) Dot spots of ten-fold dilutions of MRSA culture when exposed to hybrid MNs loaded with different concentrations of VAN (0, 0.5, 1, 10 mg g^−1^) and additionally exposed to NIR irradiation (+). Dot spots were prepared after (d) 4 h or (e) 24 h of incubation of samples after treatment end. (f) Quantification of CFU from bacterial samples after treatment with hybrid MN arrays with NIR laser irradiation for 15 min.

Finally, we analyzed the antibacterial effects of the hybrid MN arrays by exposing planktonic MRSA to VAN-loaded MN arrays with and without near-IR laser irradiation. Fig. 6a shows an illustration of the experimental setup, in which we added 200 μL of MRSA solution to the hybrid MN arrays in a well plate that was irradiated from the top with an 808 nm laser at 1 W cm^−2^ for 15 min. The increase in heat was monitored underneath the well plate using a thermal camera. Examples of the infrared images obtained with the thermal camera are shown in Fig. 6b after 5 and 15 min of irradiation. The temperature was measured at two different locations in the center (circle) and on the edge (triangle) of the well plate. The average of 36 pixels around the two measurement points as a function of time is plotted in Fig. 6c. The temperature increased upon laser irradiation, reaching a maximum of approximately 50 °C after 15 min, and we can maintain the temperature in the therapeutic heat range of 45 to 55 °C for 10 min at the center of the sample. Upon evaluating the heat profile in excised porcine skin shown in the SI, Fig. S9, we observe that such therapeutic temperature increase can be maintained also in skin tissue. However, we found that the MN array did not evenly heat the whole sample, likely because of its smaller size compared to the well plate, with the temperature at the edge of the well increasing to the therapeutic heat range only for 5 min. Such uneven heat profiles might negatively influence the antibacterial efficiency in our experimental setup. Moreover, these results highlight the importance of prospective studies to ensure full coverage of bacterially infected skin using MN arrays.

Finally, to study the antibacterial effect of both VAN and heat from the hybrid MN arrays, we fabricated hybrid MNs with different VAN concentrations (0, 0.5, 1, and 10 mg g^−1^) and exposed them to MRSA with and without near-IR laser irradiation for 15 min. After treatment, we incubated the bacterial solutions for 4 or 24 h and quantified the surviving bacteria by counting CFUs in serial dilutions. Fig. 6d-e shows top-view images of the serial dilutions of the samples on agar plates, and the quantification of the bacteria (CFU mL^−1^) is shown in Fig. 6d. We observed high bacterial growth in the untreated samples, reaching 10^9^ and 10^10^ CFU mL^−1^ after 4 and 24 h, respectively, which is well above the bacterial manifestation found in clinical skin infections [80]. In the absence of VAN (0 mg g^−1^) and the presence of only near-IR laser irradiation for 15 min (Fig. 6d, filled symbols), there was no difference in bacterial growth when compared to the control conditions (0 mg g^−1^ VAN and without laser, Fig. 6d, open symbols), indicating that the temperature increase itself does not affect MRSA growth. In the absence of laser irradiation (open symbols), increasing the VAN concentration inhibited bacterial growth, indicating the successful release of VAN from the hybrid MN arrays into the solution. After 4 h of incubation (Fig. 6d, squares), increasing the VAN concentration from 1 to 10 mg g^−1^ did not further decrease bacterial survival, although a drastic growth reduction of 5.7 log was achieved after 24 h of incubation (Fig. 6d, circles). Such an increased reduction of bacterial growth over a longer incubation time supports the time rather than the concentration-dependent action of VAN [81]. Upon analyzing the antibacterial effect in terms of the bacterial survival rate in the SI, Fig. S10 we find that after 24 h the VAN concentration of 10 mg g^−1^ resulted in almost 0% MRSA survival, therefore, we did not study the antibacterial effect of higher VAN concentrations. Importantly, the addition of the laser treatment (Fig. 6d, filled symbols) improved the antibacterial effect with a maximum 2 and 1 log reduction at VAN concentrations of 0.5 and 1 mg g^−1^, respectively, indicating that this enhanced effect is attributed to a synergistic effect between the heat and the drug. Similar antibacterial effects when combining VAN with photothermal treatment have been reported in the literature; however, in this study presented here a lower, clinically relevant laser intensity [82,83] and treatment duration was required [82]. Finally, we measured the intradermal delivery of VAN after application of hybrid MNs arrays to full-thickness porcine skin under near-IR laser irradiation, confirming that we are able to deliver 9.5 µg VAN into the skin. Therefore, the hybrid MN arrays produced here show the potential to improve local antibiotic treatment of bacterial skin infections.

## 4 Conclusion

In this work, we present the fabrication of a novel hybrid design of MN arrays consisting of a water-soluble outer layer loaded with the antibiotic VAN and a water-insoluble inner core loaded with plasmonic photothermal Au/SiO_2_ NPs for the potential co-therapy of MRSA-associated infections. We studied two water-soluble polymers, PVP and PVA, at varying concentrations and their effects on the MN morphology, inner and outer layer formation, drug loading, and photothermal performance. We found that using PVP as the water-soluble polymer results in the formation of a drug-loaded MN tip, whereas using PVA results in the formation of an outer shell. Employing PVA as the polymer matrix for the outer MN layer led to increased VAN delivery and photothermal performance compared to PVP, while the mechanical strength at 30 wt% PVA was comparable to that of PVP. Furthermore, we studied the synergistic effect of VAN and heat on MRSA and found an optimal therapeutic window of 45–55 °C for 10 min treatment. Finally, we analyzed the temperature increase and antibacterial effect of the hybrid MN arrays against MRSA, which indicated that the addition of photothermal treatment improved bacterial growth inhibition. Prospective studies will evaluate the antimicrobial performance of the hybrid MN arrays in *ex* and *in vivo* studies. This work further advances the field in MN array design and drug delivery representing a significant step forward of combining the effect of heat in local antibiotic therapies of skin infections from antibiotic resistant bacteria.

## Supplementary Material

Supplementary Material

## Data Availability

Data will be made available on request.
