# Supplementary material for: Hybrid microneedle arrays for antibiotic and near-IR photothermal synergistic antimicrobial effect against Methicillin-Resistant Staphylococcus aureus
Source: Chem Eng J. Author manuscript; Available in PMC 2023 Sep 15. (PMC7615096; doi:10.1016/j.cej.2023.142127)
Supplement: Supplementary Material [file EMS187315-supplement-Supplementary_Material.pdf]

Supporting Information

Hybrid microneedle arrays for antibiotic and near-IR photothermal synergistic antimicrobial effect against Methicillin-Resistant *Staphylococcus aureus*

*Jill Ziesmer, Justina Venckute Larsson, Georgios A. Sotiriou**

Department of Microbiology, Tumor and Cell Biology, Karolinska Institutet, SE-171 77, Stockholm, Sweden

*E-mail: georgios.sotiriou@ki.se

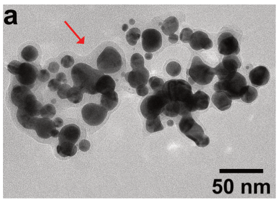

b

Batch	SSA (m²/g)	XRD 38 deg. peak (nm)
1	18.1 ± 0.4	14.7
2	17.2 ± 0.3	13.7
3	15.7 ± 0.2	11.5

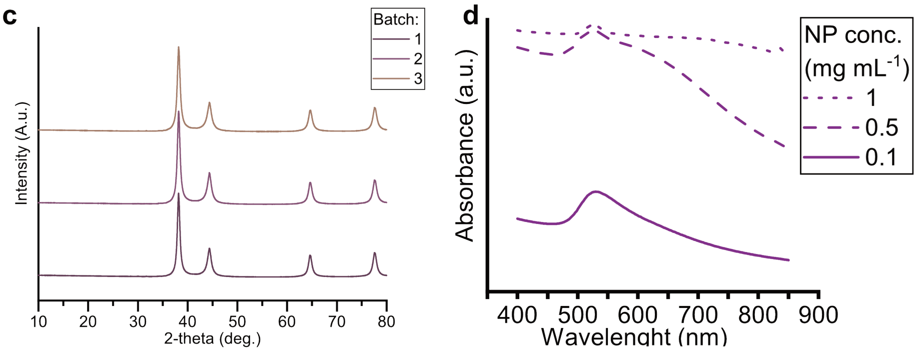

Figure S1: Summary of the characterization of Au + 4 wt% SiO_2 nanoaggregates. a) Representative TEM image of the nanoaggregate. The red arrow indicates the SiO_2 layer. b) The specific surface area (SSA) measured with N_2 absorption with Brunauer-Emmett-Teller method and the crystal size measured from the 38° peak of the XRD for three different batches of nanoparticle fabrication. c) The XRD diffractogram of nanoparticle powders from three batches. d) The UV/Vis absorbance of nanoaggregates dispersed in water at different concentrations.

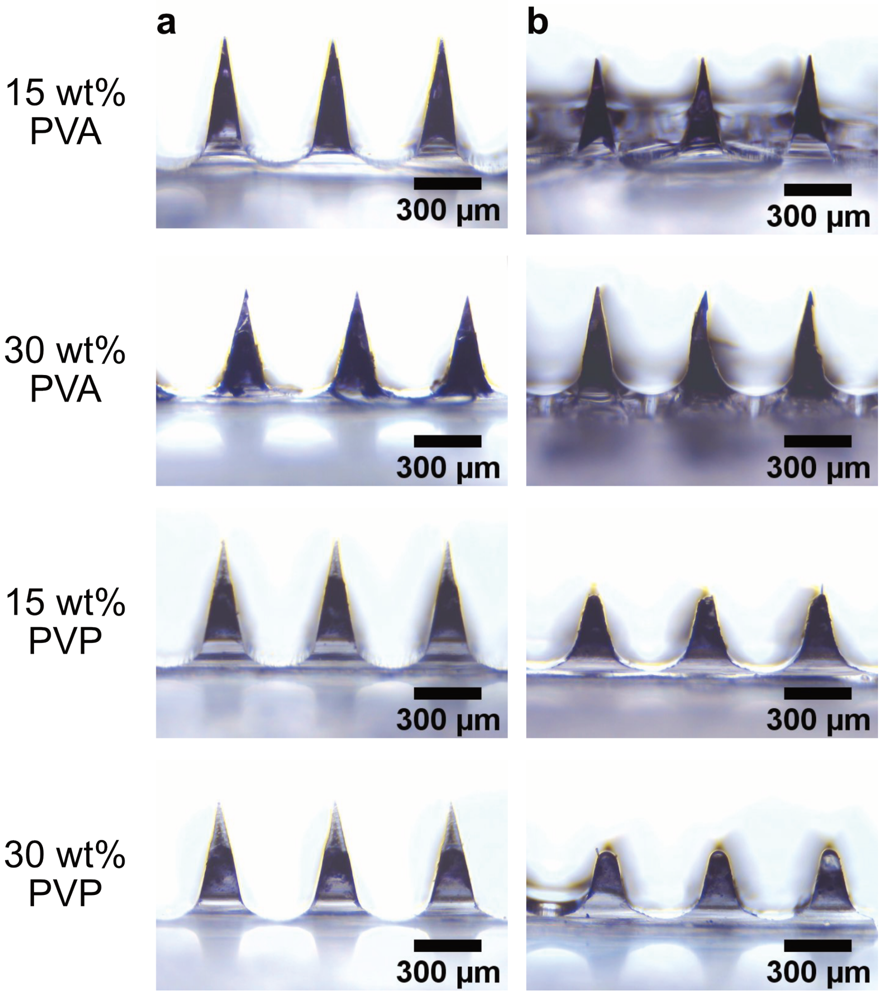

Figure S2: Bright field microscopy images of hybrid MN arrays produced with 10 mg g^{-1} VAN (a) before and (b) after dissolution in PBS for different polymers and weight percentages.

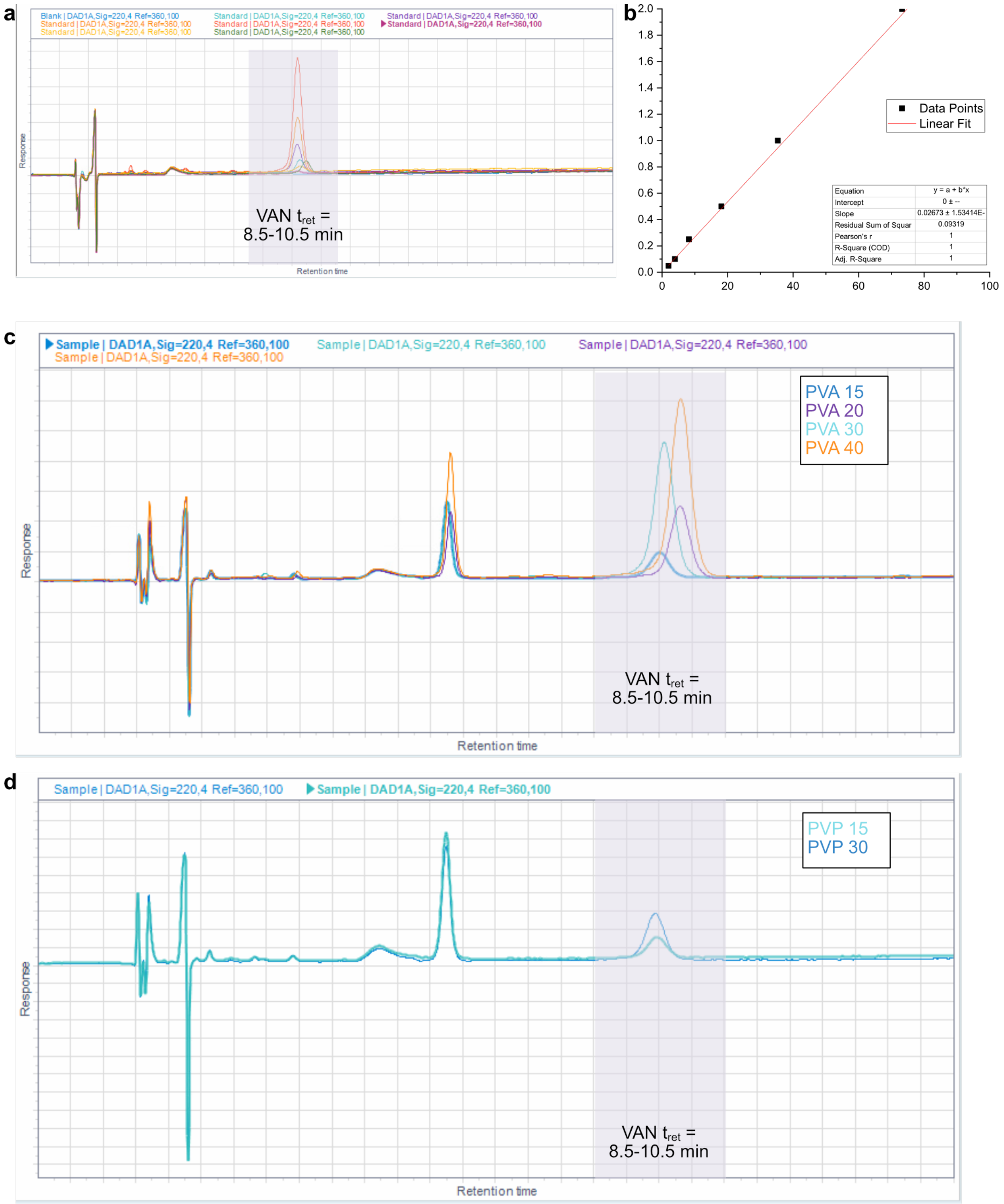

Figure S3: Raw data of the VAN quantification via HPLC. The chromatogram (a) and the linear regression of the VAN concentration and peak area (b) of the VAN standard at 0.05 – 2 µg mL^{-1}. Chromatograms of 1:10 dilutions of completely dissolved hybrid MN samples loaded with 10 mg g^{-1} VAN for varying PVA (c) and PVP (d) weight percentages.

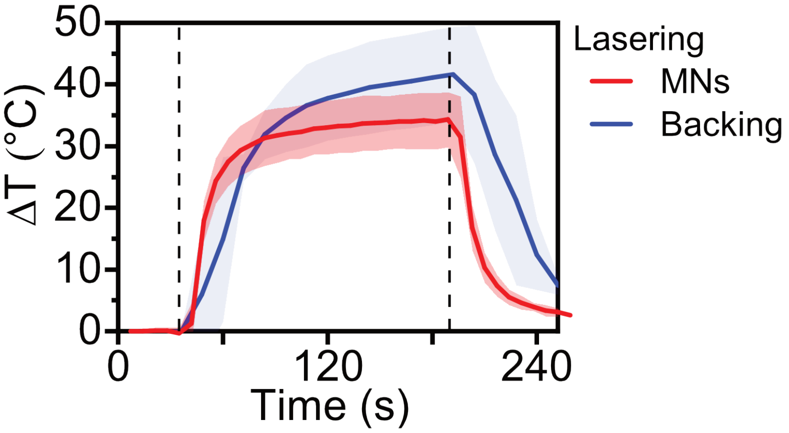

Figure S4: Photothermal heating of hybrid MN arrays (prepared with 10 mg g^{-1} VAN and 20 mg g^{-1} Au/SiO$_2$ NPs) at 808 nm laser radiation in air comparing laser irradiation when applied from the top (MNs) or the bottom (backing) of the MN array.

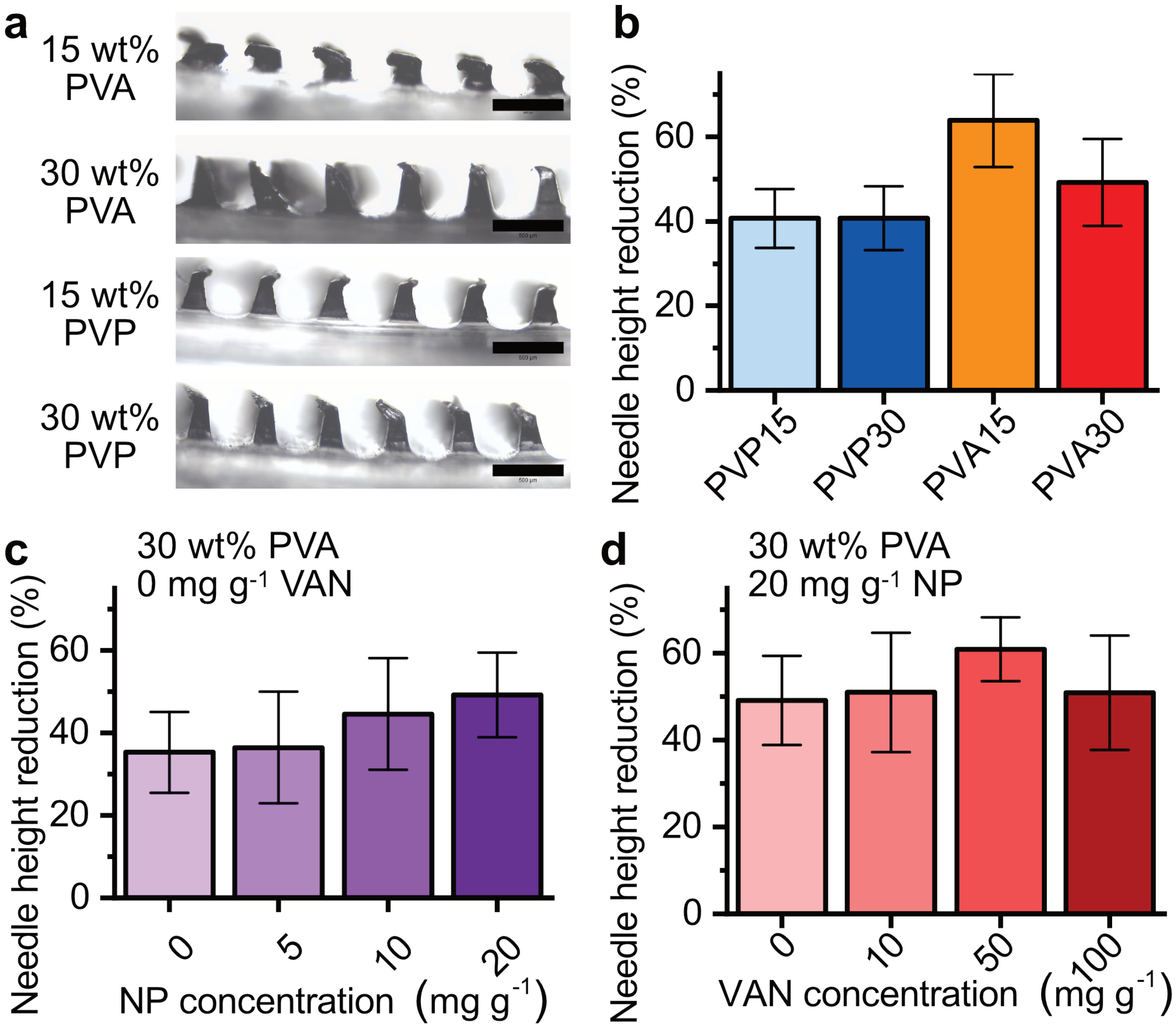

Figure S5: Mechanical testing of hybrid MN arrays. (a) Bright field microscopy images of hybrid MN arrays produced with different polymers and weight percentages after compression with a Texture Analyzer for 30 s at 3.2 kN, scale bar indicates 500 µm. The MN height reduction after compression was quantified for hybrid MN arrays produced with (a) different polymers and weight percentages, (b) for increasing NP concentration in the photothermal core, or (c) for increasing VAN concentration in the water-soluble shell. At least 10 needles were measured before and after compression, n=3.

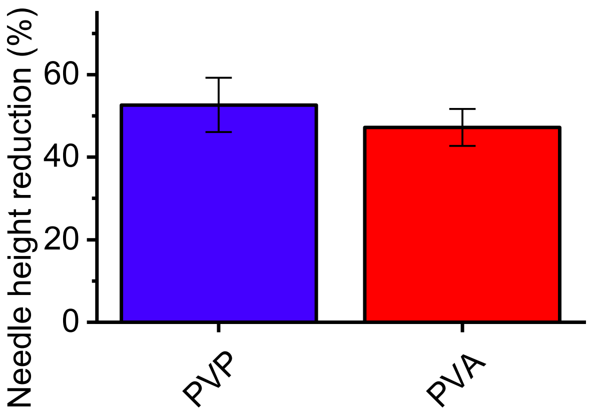

Figure S6: Needle height reduction after compression at 3.2 kN for 30 s for MN arrays made out of 30 wt% PVP or PVA.

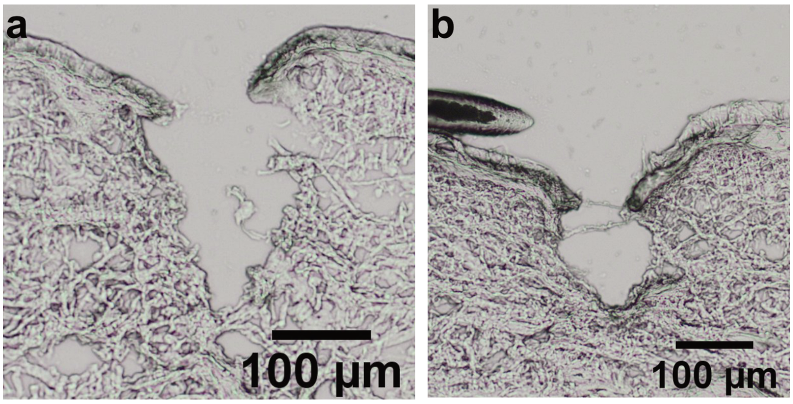

Figure S7: Bright-field microscopy images of skin tissue sections after insertion and removal of hybrid MN arrays with thumb pressure.

Growth Reduction (%)						
		VAN (ug/ mL)				
		0	0.125	0.25	0.5	0.75
Heat (deg. C), 5 min						
50	Mean	-6.73941	1.35069	2.29516	7.514	19.68355
55	Mean	15.94333	22.44941	23.9902	25.28557	26.98309
60	Mean	26.58555	39.20114	39.48519	85.41497	99.99724
50	STD	3.11067	4.46373	5.52631	5.72359	1.62856
55	STD	17.14797	14.0647	12.1827	14.37967	1.99692
60	STD	6.30812	2.49952	3.985	11.28685	0.27748
Heat (deg. C), 10 min						
50	Mean	8.29514	17.62166	19.04499	18.97424	27.41458
55	Mean	28.10476	31.48662	43.00959	72.93011	98.91517
60	Mean	31.06531	37.86265	48.89678	77.75386	99.98352
50	STD	6.79605	5.71148	5.65978	5.65646	11.03156
55	STD	2.06225	1.45255	3.62232	21.94516	0.84874
60	STD	6.35042	6.28233	17.54514	38.46947	0.33005
Heat (deg. C), 15 min						
50	Mean	1.81621	6.9672	8.92112	11.45264	29.27751
55	Mean	25.48235	32.38912	43.60536	62.01769	99.54403
60	Mean	23.48528	37.0117	61.02355	94.90324	100.0621
50	STD	5.07774	3.72184	4.56019	4.01066	5.81962
55	STD	2.10663	5.67614	4.62969	32.27014	0.44725
60	STD	9.22282	6.36915	33.95229	8.64115	0.31509

Figure S8: Raw data of the growth inhibition for synergistic treatment between VAN and heat as shown in Figure 5a of the main text.

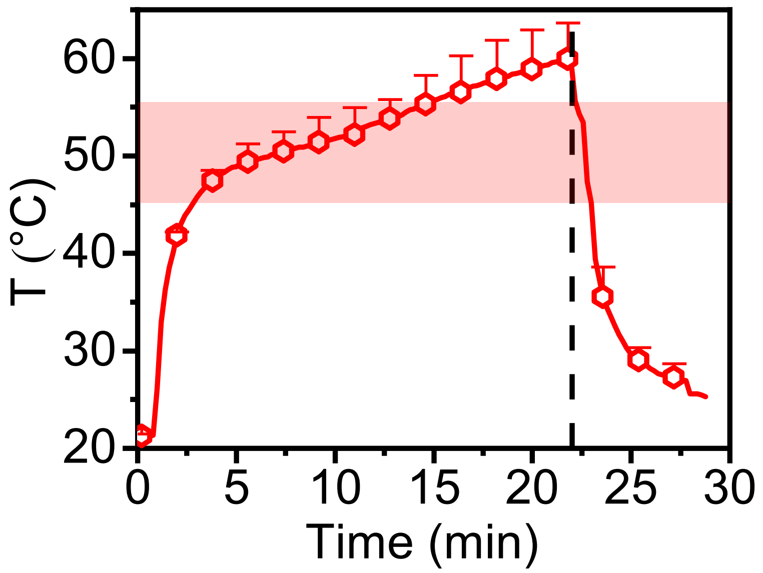

Figure S9: Temperature increase over time for application of hybrid MN arrays (prepared with 20 mg g^{-1} Au/SiO$_2$ NPs) into excised porcine skin under near-IR irradiation at 1 W cm^{-2} at 808 nm.

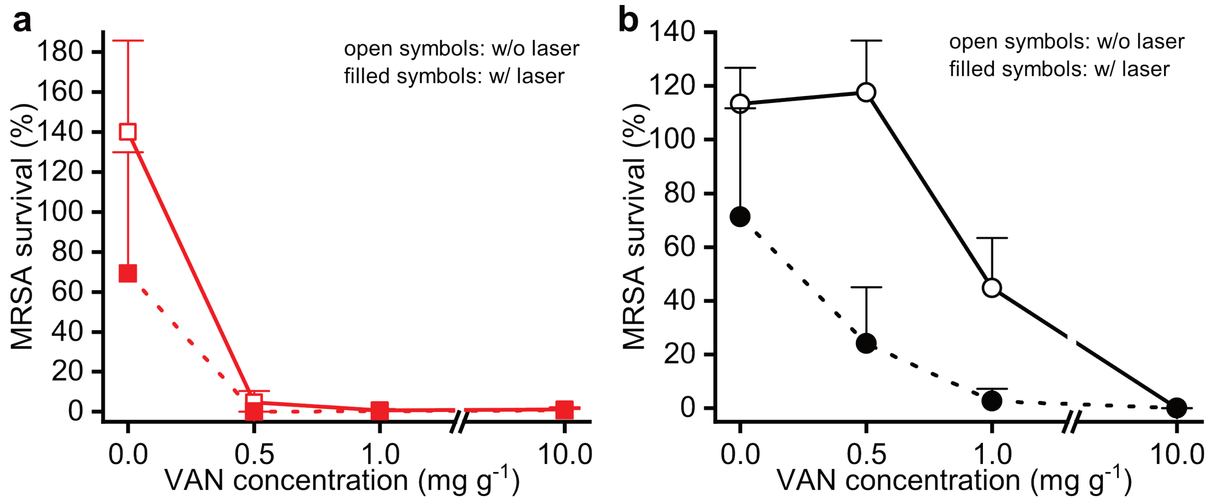

Figure S10: Survival rate of MRSA after treatment with hybrid MN arrays with or without near-IR irradiation at 808 nm and 1 W cm^{-2} after 4 h (a) or 24 h (b) of incubation after treatment.
